# Supplementary material for: LipidIN: a comprehensive repository for flash platform-independent annotation and reverse lipidomics
Source: Nat Commun. 2025 May 16;16:4566. doi: 10.1038/s41467-025-59683-5 (PMC12084368; doi:10.1038/s41467-025-59683-5)
Supplement: Supplementary file 1 — Supplementary Information [file 41467_2025_59683_MOESM1_ESM.pdf]

Supporting Information

LipidIN: a comprehensive repository for flash platform-independent annotation and reverse lipidomics

Hao Xu[1,2,12], Tianhang Jiang[1,12], Yuxiang Lin[3,12], Lei Zhang[1,12], Huan Yang[1,4], Xiaoyun Huang[5], Ridong Mao[1], Zhu Yang[6], Changchun Zeng[7], Shuang Zhao[8], Lijun Di[9], Wenbin Zhang[10], Jun Zeng[5,*], Zongwei Cai[6,11,*], Shu-Hai Lin[1,2,*]

[1] The First Affiliated Hospital of Xiamen University, State Key Laboratory of Cellular Stress Biology, School of Life Sciences, XMU-HBN skin biomedical research center, Xiamen University, Xiamen, Fujian 361102, China.

[2] School of Medicine, National Institute for Data Science in Health and Medicine, Xiamen University, Xiamen, Fujian 361102, China.

[3] Department of Breast Surgery, Fujian Medical University Union Hospital, 350001, Fuzhou, Fujian Province, China.

[4] School of Pharmaceutical Sciences, Xiamen University, Xiamen, Fujian 361102, China.

[5] College of Ocean Food and Biological Engineering, Jimei University, Xiamen 361021, China.

[6] State Key Laboratory of Environmental and Biological Analysis, Department of Chemistry, Hong Kong Baptist University, Kowloon 999077, Hong Kong, China.

[7] Department of General Medicine, Shenzhen Longhua District Central Hospital, Shenzhen, China.

[8] Xiamen Meliomics Co., Ltd., Xiamen, Fujian 361026, China.

[9] Department of Biological Sciences, Faculty of Health Sciences, University of Macau, Macau, China.

[10] Department of Occupational and Environmental Health and the Ministry of Education Key Lab of Hazard Assessment and Control in Special Operational Environment, School of Public Health, Fourth Military Medical University, Xi'an, China.

[11] Eastern Institute of Technology, Ningbo 315200, China

[12] These authors contributed equally to this work.

To whom correspondence should be addressed: Jun Zeng (E-mail: junzeng@jmu.edu.cn), Zongwei Cai (E-mail: zwcai@hkbu.edu.hk), Shu-Hai Lin (E-mail: shuhai@xmu.edu.cn)

Supplementary Figures

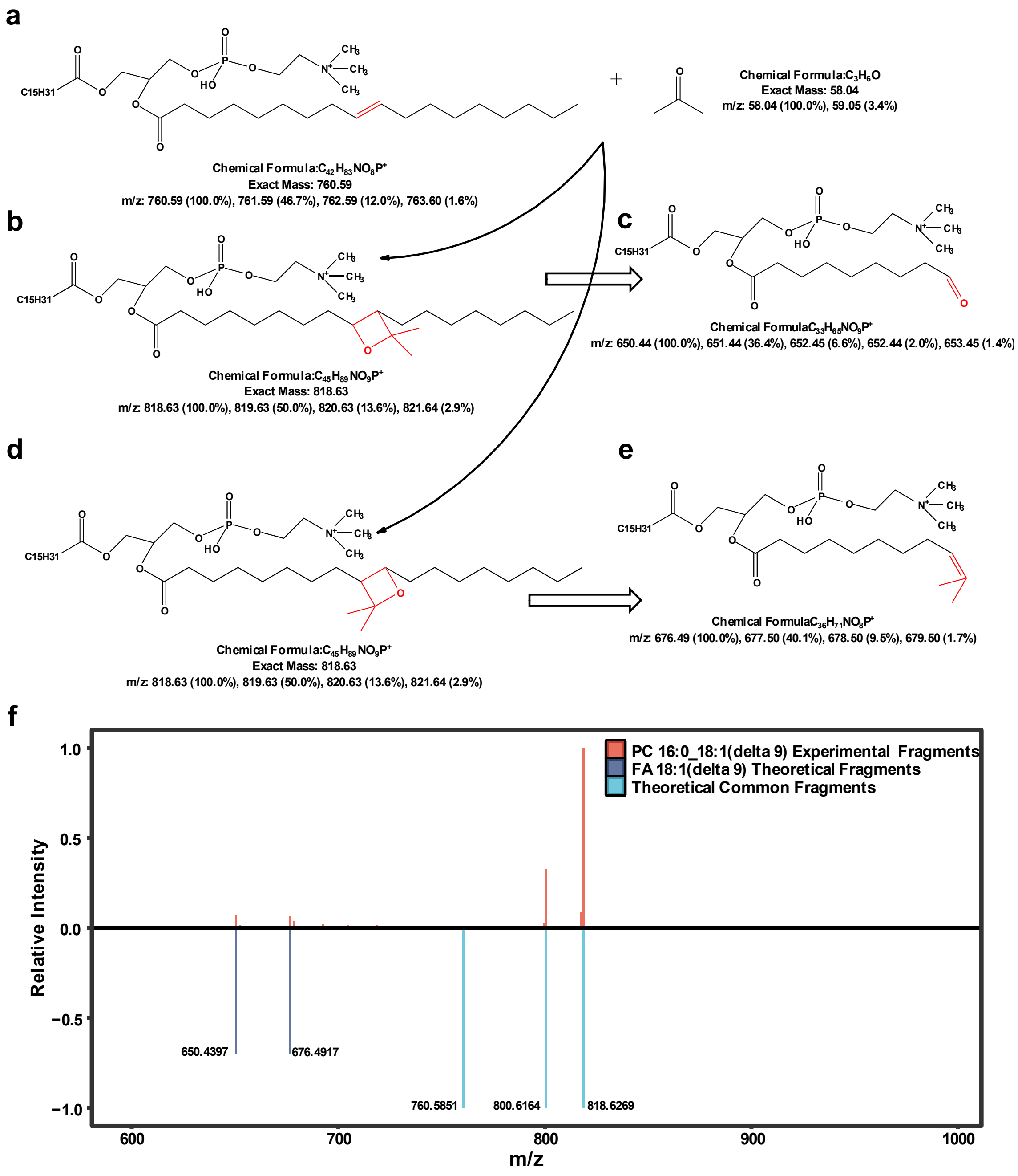

Supplementary Fig. 1. Schematic representation of Paternò-Büchi reaction with acetone and MS^2 CID of a glycerophospholipid. a. Structural formula for PC 16:0_18:1 (delta 9), $[M+H]^+$, using red color to mark the C=C position. **b, d.** An isomer produced by the reaction of PC 16:0_18:1 (delta 9) with acetone. **c, e.** Breakage rules for isomers in mass spectrometry. **f.** The red color shows the spectrum of PC 16:0_18:1 (delta 9) in one experiment. The purple color represents the paired break peaks (650.4397, 676.4317) representing C18:1 (delta 9) in CID mode. The blue color represents the molecular ion peak (818.6269) after the reaction of PC 16:0_18:1 (delta 9), the neutral loss peak (800.6164), and the characteristic peak of the three methyl groups in the lost head group (760.5851).

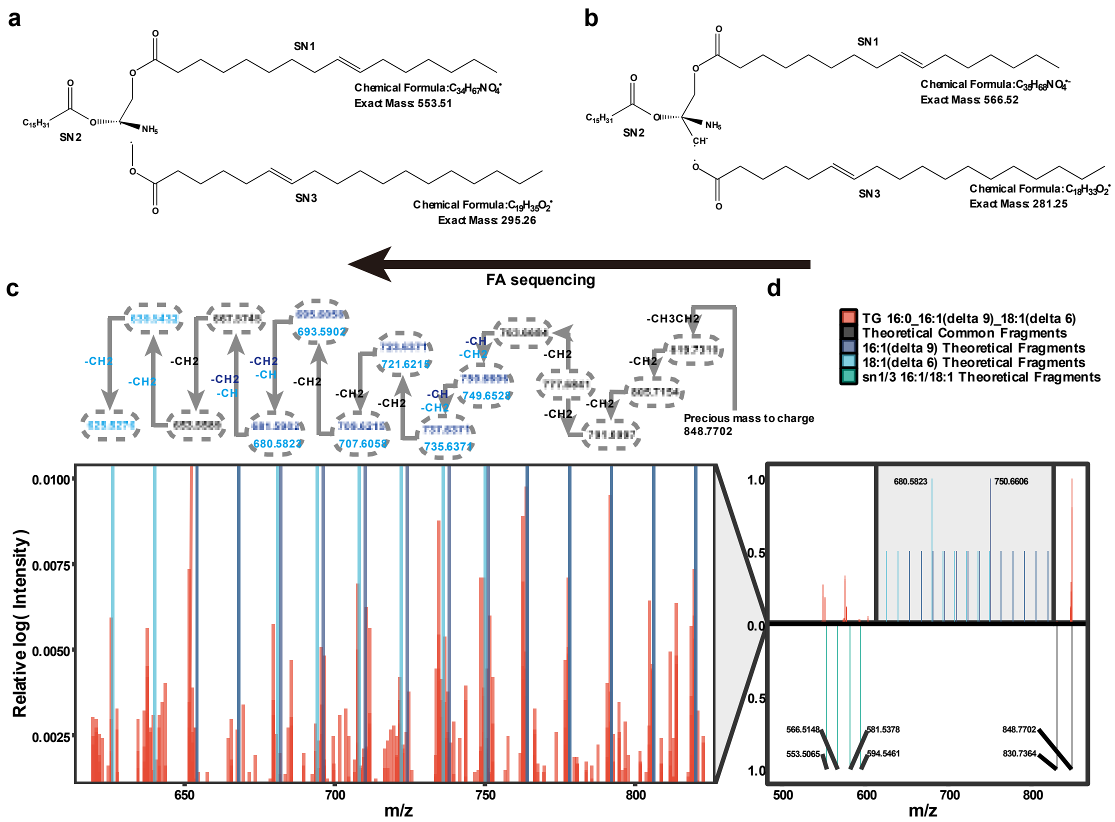

Supplementary Fig. 2. Schematic representation of electron-activated dissociation (EAD) of a Triglyceride. a-b. A breakage scenario of C18:1 (delta 6) at sn1/3 position in EAD mode for TG 16:0_16:1(delta 9)_18:1(delta 6), $[M+NH_4]^+$. **c.** The resulting fragments are color-coded as follows: purple for C16:1(delta 9), blue for C18:1(delta 6). The corresponding carbon loss is specified above each arrow using the same color code. Carbon losses can be $-CH_3CH_2$ (25 Da), $-CH_2$ (14 Da), -CH (13 Da). **d.** The resulting fragments are color-coded as follows: red for spectrum of TG 16:0_16:1(delta 9)_18:1(delta 6), $[M+NH_4]^+$ in one experiment, black for common fragments (molecular ion peak: 848.7702, NL of molecular ion peak: 830.7364), green for sn1/3 C16:1/C18:1.

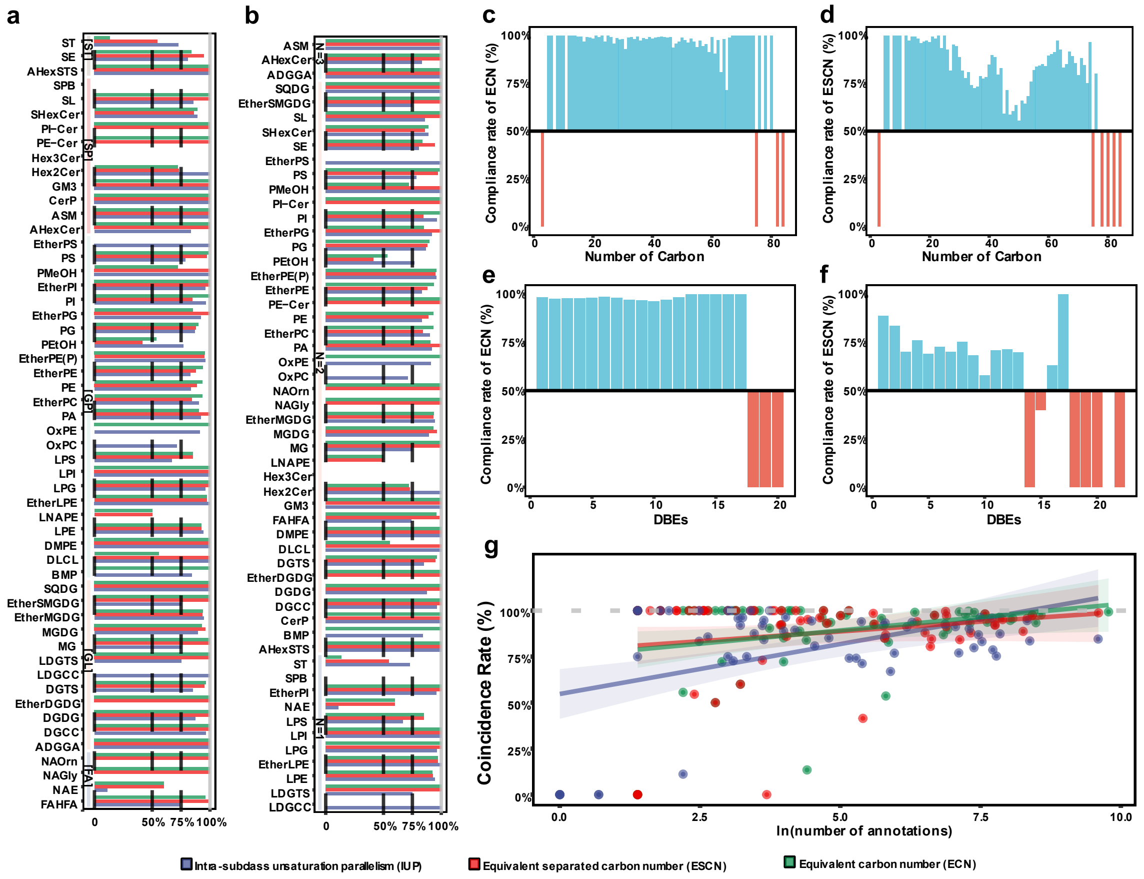

Supplementary Fig. 3. Detail Relative retention time rules of lipid subclasses. a-b. Statistical results of ECN, IUP, ESCN for 101 public datasets, covering 54 subclasses, classified by different lipid category and number of separated chains. **c-f.** Statistical results of ECN, IUP, ESCN, classified by different number of carbons and poly-unsaturations. **g.** Fitting the relationship between the number of annotations and the degree of conformity.

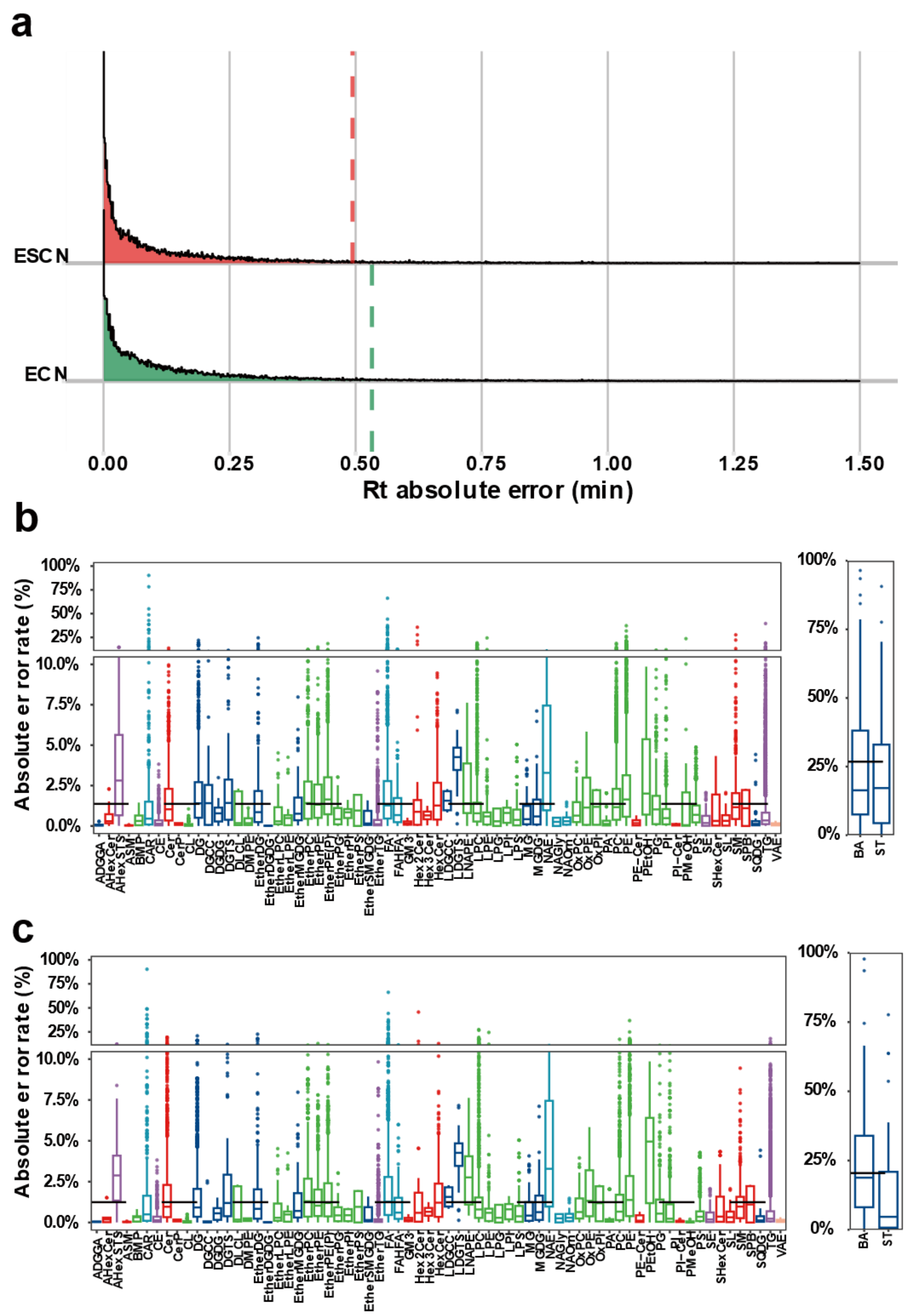

Supplementary Fig. 4. Display of error values and error rates for ECN and ESCN in 101 published dataset. a. Absolute retention time error statistics: red for ECNs, blue for ESCNs, with a dotted line representing the 95% quantile. **b-c.** Box plots of absolute retention time deviation rates for ECN and ESCN by lipid subclass, with the black horizontal line indicating the population mean, in the box plot the median as a center line, with the box representing the interquartile range (IQR) between the upper and lower quartiles. Whiskers extend to 1.5 times the IQR, and points beyond this range indicate outliers.

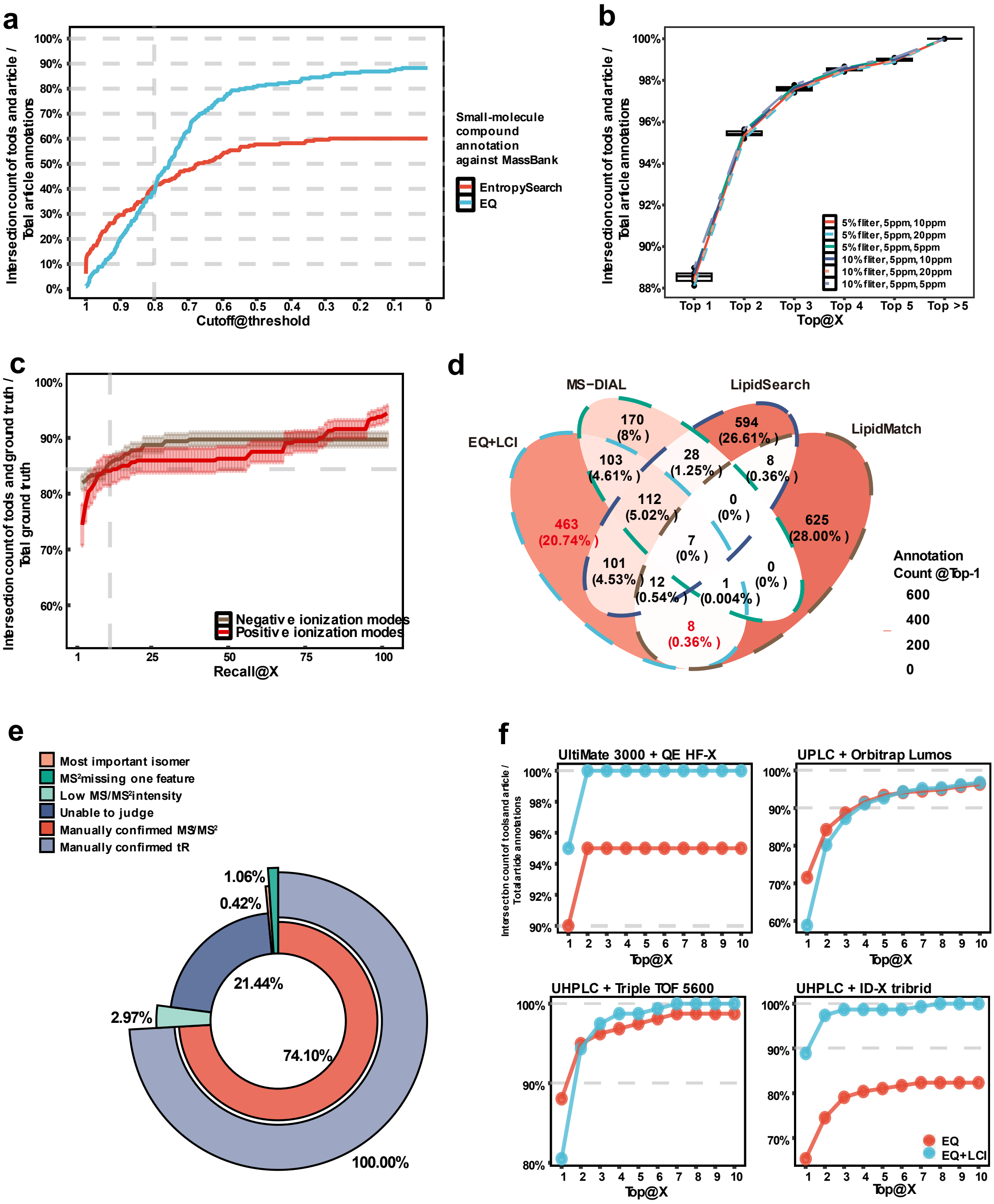
a
Intersection count of tools and article / Total article annotations
Cutoff@threshold
Small-molecule compound annotation against MassBank
EntropySearch
EQ
b
Intersection count of tools and article / Total article annotations
Top@X
Top 1
Top 2
Top 3
Top 4
Top 5
Top >5
5% fliter, 5ppm, 10ppm
5% fliter, 5ppm, 20ppm
5% fliter, 5ppm, 5ppm
10% fliter, 5ppm, 10ppm
10% fliter, 5ppm, 20ppm
10% fliter, 5ppm, 5ppm
c
Intersection count of tools and ground truth / Total ground truth
Recall@X
Negative ionization modes
Positive ionization modes
d
MS−DIAL
LipidSearch
LipidMatch
EQ+LCI
170 (8%)
594 (26.61%)
103 (4.61%)
28 (1.25%)
8 (0.36%)
112 (5.02%)
0 (0%)
463 (20.74%)
625 (28.00%)
7 (0%)
101 (4.53%)
12 (0.54%)
1 (0.004%)
0 (0%)
8 (0.36%)
Annotation Count @Top-1
600
400
200
0
e
Most important isomer
MS²missing one feature
Low MS/MS²intensity
Unable to judge
Manually confirmed MS/MS²
Manually confirmed tR
1.06%
0.42%
21.44%
2.97%
74.10%
100.00%
f
Intersection count of tools and article / Total article annotations
UltiMate 3000 + QE HF-X
UPLC + Orbitrap Lumos
UHPLC + Triple TOF 5600
UHPLC + ID-X tribrid
Top@X
EQ
EQ+LCI

Supplementary Fig. 5. Additional comparison of EQ plus LCI. a. Recall rates of EQ+LCI and EntropySearch in small-molecule compound annotation. **b.** The library querying was performed using the EQ module with the MS1 tolerance set at 5 and the MS2 tolerance set at 5,10 or 20 when the Orbitrap instrument spectral was filtered for the highest 10% or 5% of peaks, respectively, in the box plot the median as a center line, with the box representing the interquartile range (IQR) between the upper and lower quartiles. Whiskers extend to 1.5 times the IQR, and points beyond this range indicate outliers. **c.** Recall@ Top-100 of lipids without MS^2 prediction in both ionization modes, by performing LCI module using 10-fold random sampling, in the plot the median as a center point, with the box representing the interquartile range (IQR) between the upper and lower quartiles. Whiskers extend to 1.5 times the IQR, and points beyond this range indicate outliers. **d.** Venn diagram of common annotation@Top-1 by four methods: MS-DIAL, LipidSearch, EQ+LCI, and LipidMatch. **e.** 471 lipids validated by manually checking MS/MS fragmentation and agreement with relative retention time by ECN, IUP, ESCN rule. In the final check, "Manually confirmed" indicates that both MS2 and retention time rules are in agreement, "MS2 loss" indicates that some of the secondary spectrum peaks are missing, but the identification is still correct, "better" means that there is a better annotation to choose from (contained in the intersection of multiple methods), but this annotation turns out to be correct, "Low MS1/MS2 intensity" indicates that the primary or secondary spectral response of the annotation has a low intensity, "Unable to judge" means that the we cannot guarantee that the annotation is completely correct, but there is no evidence that the annotation is wrong, "Manual confirmation and retention time deviation within thresholds" indicates that within the retention time threshold of 0.5 min, the identification result can be considered to be consistent with the secondary spectrum and the retention time rule. **f.** EQ and EQ+LCI performance in the datasets acquired from different LC-MS systems for Recall@ Top-10, respectively.

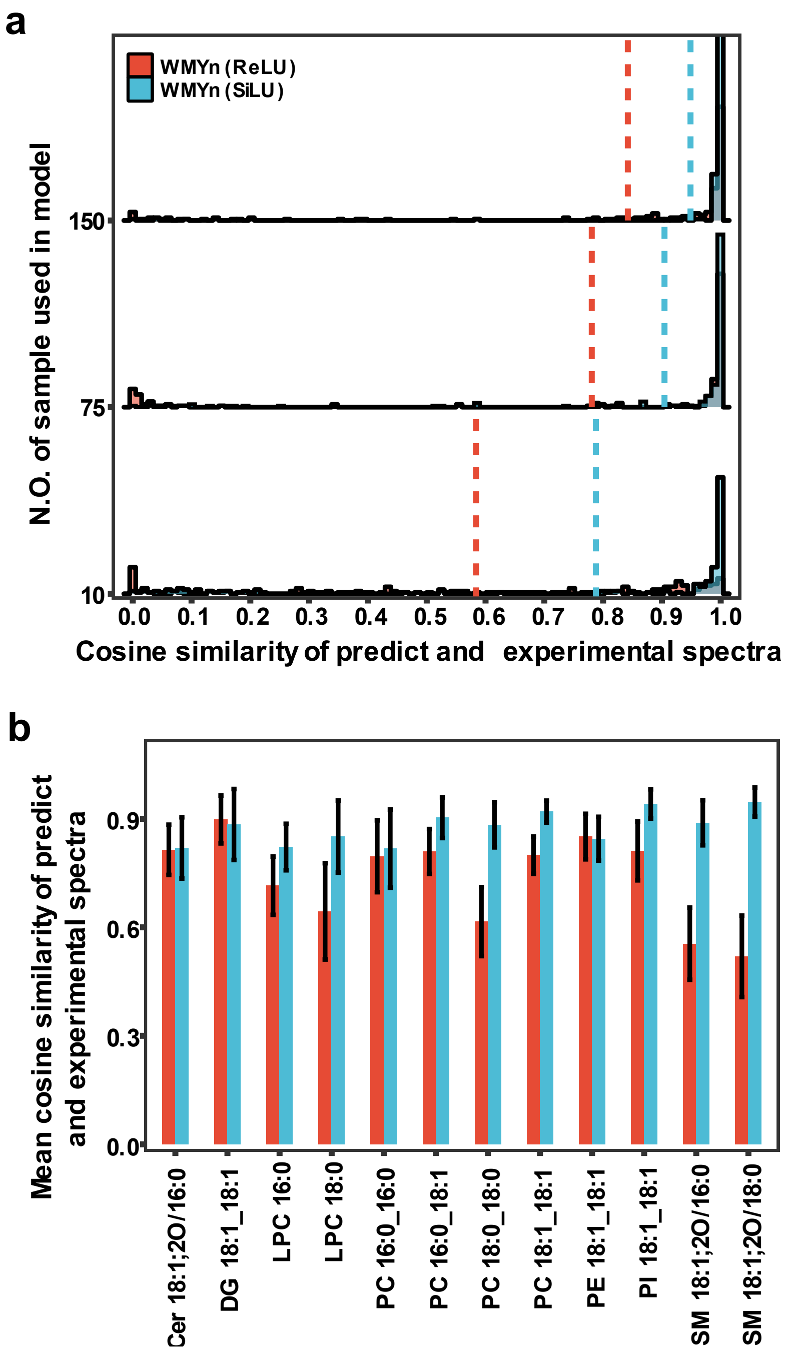

Supplementary Fig. 6. Cosine similarity performance comparison between WMYn (SiLU) and WMYn (ReLU). a. The cosine similarity between predicted spectra trained with different sample sizes and experimental detection spectra. The red bars represent WMYn (ReLU), the blue bars represent WMYn (SiLU), and the dotted line indicates the mean cosine similarity. **b.** The cosine similarity between the predicted spectra and experimental spectra for 15 lipid reference standards under epoch = 3000, with an input sample size of 10. Error bars represent standard deviation.

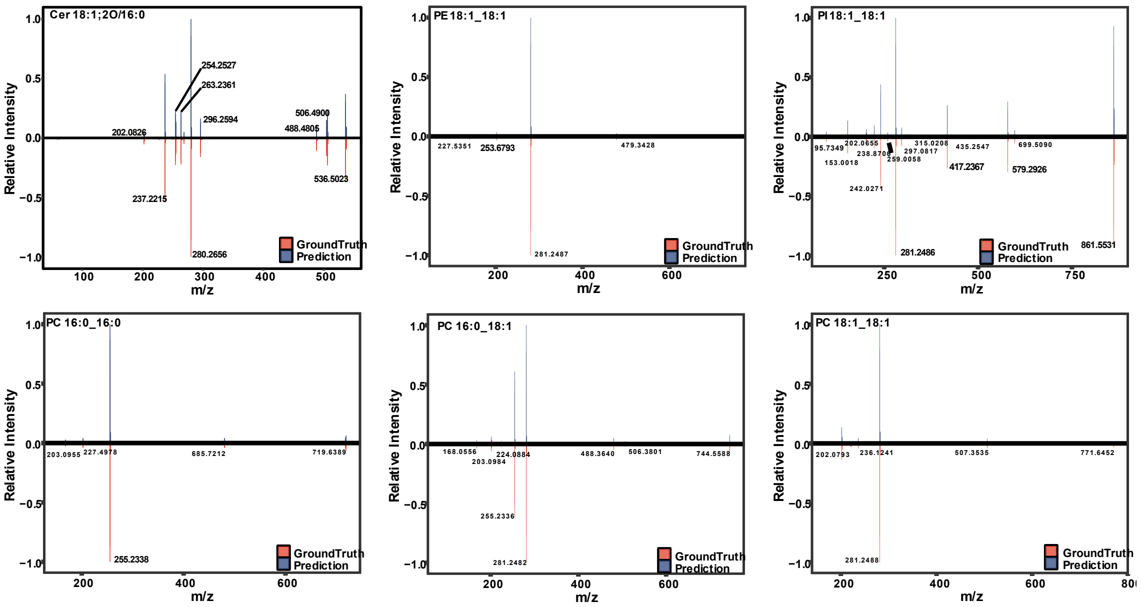

Supplementary Fig. 7. Positive ionization mode predictions for the spectrum of Orbitrap Exploris 240 MS using WMYn.

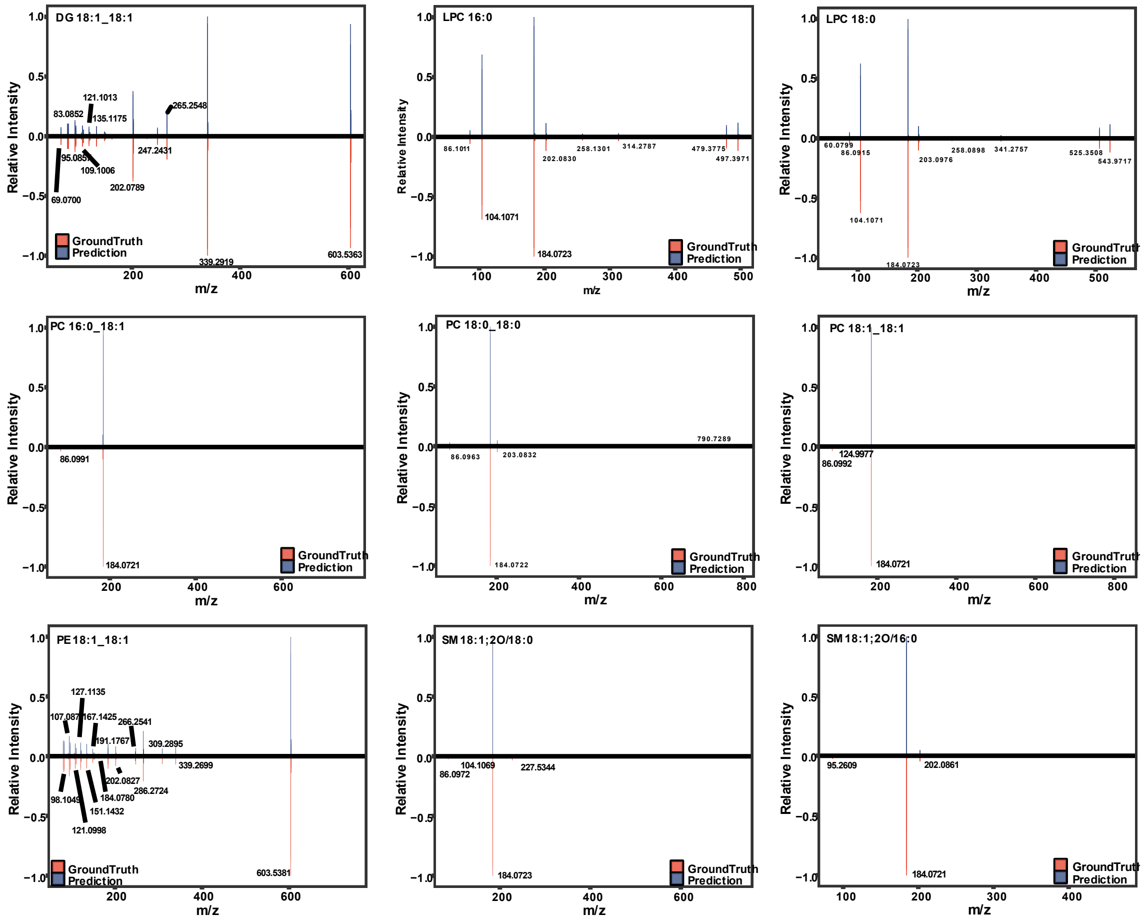

Supplementary Fig. 8. Negative ionization mode predictions for the spectrum of Orbitrap Exploris 240 MS using WMYn.

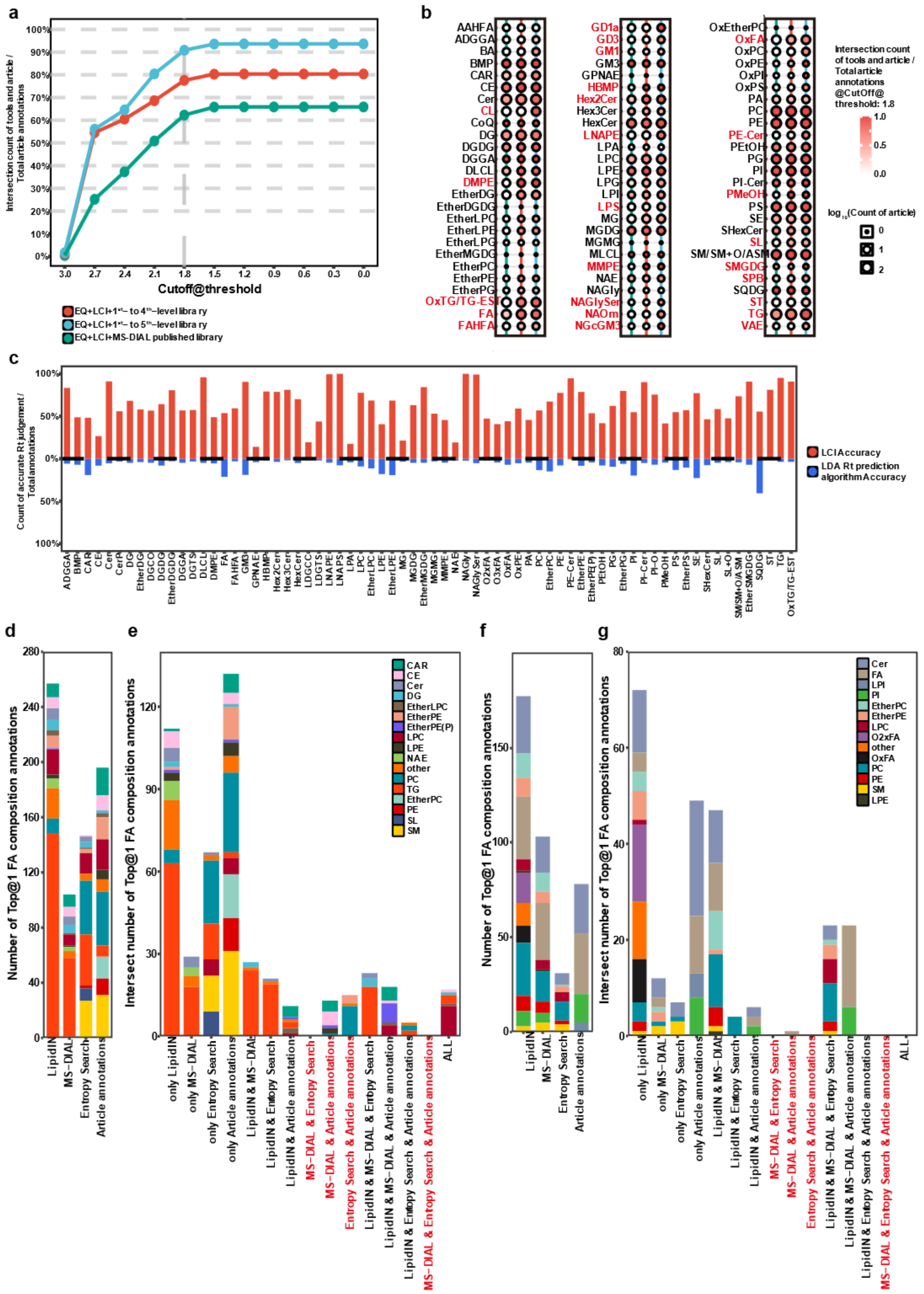
a
Intersection count of tools and article / Total article annotations
Cutoff@threshold
EQ+LCI+1st– to 4th–level library
EQ+LCI+1st– to 5th–level library
EQ+LCI+MS-DIAL published library
b
Intersection count of tools and article / Total article annotations @CutOff@ threshold: 1.8
log10(Count of article)
c
Count of accurate Rt judgement / Total annotations
LCI Accuracy
LDA Rt prediction algorithm Accuracy
d
Number of Top@1 FA composition annotations
LipidIN
MS–DIAL
Entropy Search
Article annotations
e
Intersect number of Top@1 FA composition annotations
only LipidIN
only MS–DIAL
only Entropy Search
only Article annotations
LipidIN & MS–DIAL
LipidIN & Entropy Search
LipidIN & Article annotation
MS–DIAL & Entropy Search
MS–DIAL & Article annotations
Entropy Search & Article annotations
LipidIN & MS–DIAL & Entropy Search
LipidIN & MS–DIAL & Article annotation
LipidIN & Entropy Search & Article annotations
MS–DIAL & Entropy Search & Article annotations
ALL
f
Number of Top@1 FA composition annotations
g
Intersect number of Top@1 FA composition annotations

Supplementary Fig. 9. Establishment of aging-associated lipidome atlas in mice and NIST SRM 1950. a. recall of the reported 2704 lipids in the MS-DIAL public database, 1- to 4- level hierarchical library, and 1- to 5- level library, respectively. **b.** Detial statistics of recall@cutoff@thresold 1.8. The size of the circle reflects the number of annotations in lipid subclass, and the color shade reflects the recall. Hierarchical library and reverse lipidomics demonstrated advantages in the annotation of various lipid subclasses. **c.** Comparison of Lipid Categories Intelligence Modeling (LCI) and Lipid Data Analyzer (LDA) for removal of false positive annotations in aging-associated lipidome atlas in mice. The SRM 1950 was analyzed using LipidIN, MS-DIAL, Entropy Search, and article annotations (Lipid Hunter + LipidAnnotator + manual checks). **d.** The number of Top@1 annotations for different fatty acid (FA) compositions by each method in positive ionization mode. **e.** The number of intersections of FA compositions annotated by different methods in positive ionization mode. **f.** The number of Top@1 annotations for different FA compositions by each method in negative ionization mode. **g.** The number of intersections of FA compositions annotated by different methods in negative ionization mode.

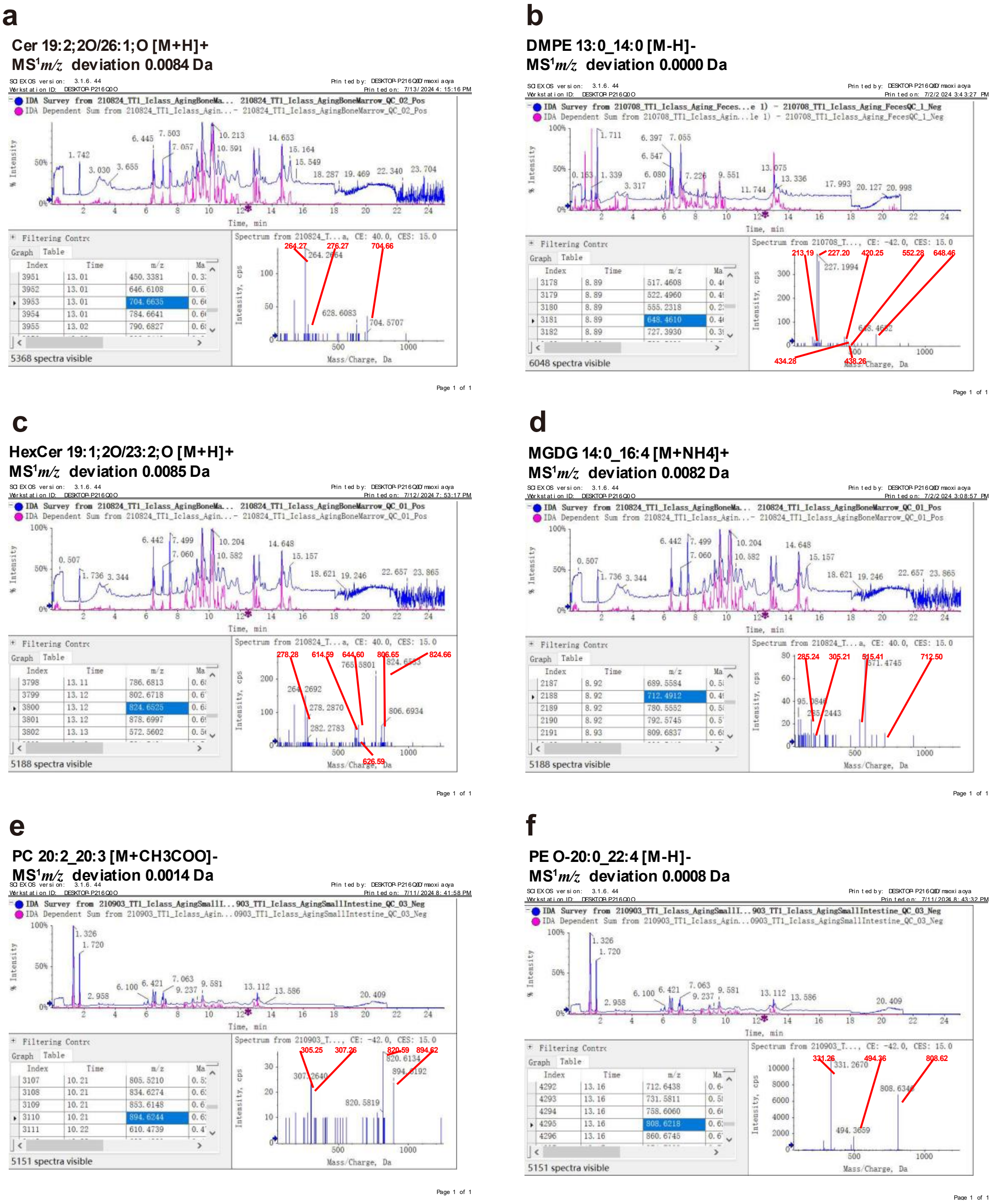
a
Cer 19:2;2O/26:1;O [M+H]+
MS1 m/z deviation 0.0084 Da
b
DMPE 13:0_14:0 [M-H]-
MS1 m/z deviation 0.0000 Da
c
HexCer 19:1;2O/23:2;O [M+H]+
MS1 m/z deviation 0.0085 Da
d
MGDG 14:0_16:4 [M+NH4]+
MS1 m/z deviation 0.0082 Da
e
PC 20:2_20:3 [M+CH3COO]-
MS1 m/z deviation 0.0014 Da
f
PE O-20:0_22:4 [M-H]-
MS1 m/z deviation 0.0008 Da

Supplementary Fig. 10. 1-6 lipids mass spectrometric spectra identified by LipidIN but not showed in the article (Raw data obtained from Tsugawa H, *et al.* A lipidome landscape of aging in mice. *Nat Aging*. 2024). a. Experimental spectra of Cer 19:2;2O/26:1;O (ion adduct in $[M+H]^+$), and labeling of MS/MS characteristic peaks. **b.** Experimental spectra of DMPE 13:0_14:0 (ion adduct in $[M-H]^-$), and labeling of MS/MS characteristic peaks. **c.** Experimental spectra of HexCer 19:1;2O/23:2;O (ion adduct in $[M+H]^+$), and labeling of MS/MS characteristic peaks. **d.** Experimental spectra of MGDG 14:0_16:4 (ion adduct in $[M+NH_4]^+$), and labeling of MS/MS characteristic peaks. **e.** Experimental spectra of PC 20:2_20:3 (ion adduct in $[M+CH_3COO]^-$), and labeling of MS/MS characteristic peaks. **f.** Experimental spectra of PE O-20:0_22:4 (ion adduct in $[M-H]^-$), and labeling of MS/MS characteristic peaks.

a

PI O-13:0_20:4 [M-H]-
MS^1 *m/z* deviation 0.0128 Da

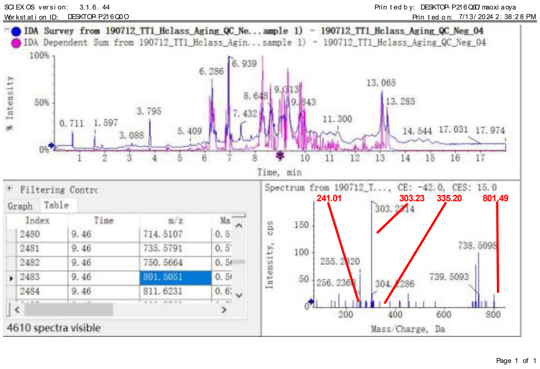

b

TG O-17:0_17:1_17:1 [M+NH4]+
MS^1 *m/z* deviation 0.0025 Da

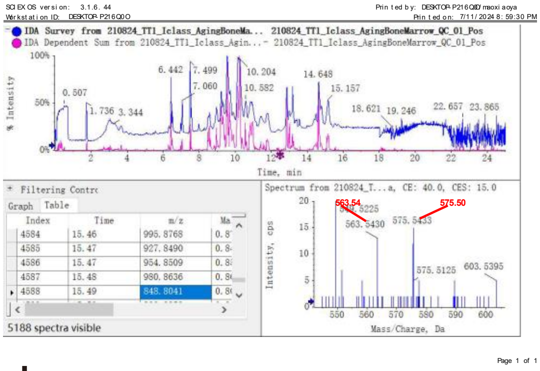

c

FA 14:3 [M-H]-
MS^1 *m/z* deviation 0.0012 Da

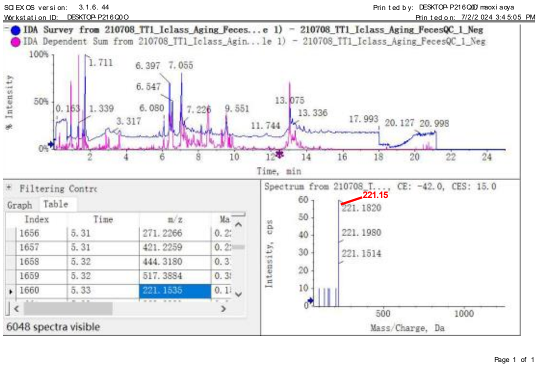

d

PG O-15:1_16:0 [M-H]-
MS^1 *m/z* deviation 0.0010 Da

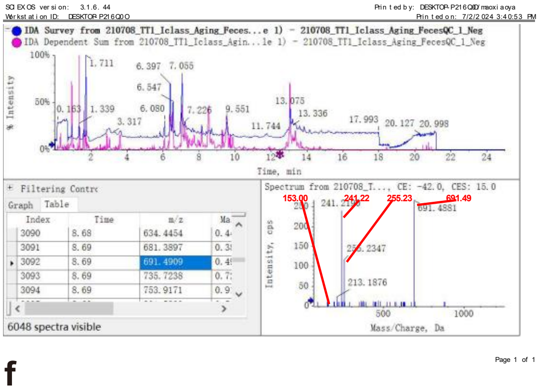

e

OxPE 16:0_20:3;O [M-H]-
MS^1 *m/z* deviation 0.0073 Da

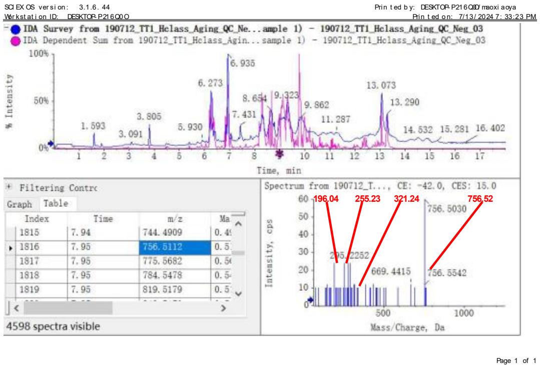

f

TG 12:0_14:0_18:2 [M+NH4]+
MS^1 *m/z* deviation 0.0012 Da

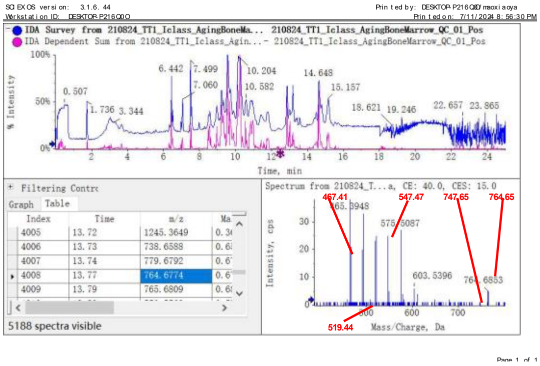

Supplementary Fig. 11. 7-12 lipids mass spectrometric spectra identified by LipidIN but not showed in the article (Raw data obtained from Tsugawa H, *et al.* A lipidome landscape of aging in mice. *Nat Aging*. 2024). a. Experimental spectra of PI O-13:0_20:4 (ion adduct in $[M-H]^-$), and labeling of MS/MS characteristic peaks. **b.** Experimental spectra of TG O-17:0_17:1_17:1 (ion adduct in $[M+NH_4]^+$), and labeling of MS/MS characteristic peaks. **c.** Experimental spectra of FA 14:3 (ion adduct in $[M-H]^-$), and labeling of MS/MS characteristic peaks. **d.** Experimental spectra of PG O-15:1_16:0 (ion adduct in $[M-H]^-$), and labeling of MS/MS characteristic peaks. **e.** Experimental spectra of OxPE 16:0_20:3;O (ion adduct in $[M-H]^-$), and labeling of MS/MS characteristic peaks. **f.** Experimental spectra of TG 12:0_14:0_18:2 (ion adduct in $[M+NH_4]^+$), and labeling of MS/MS characteristic peaks.

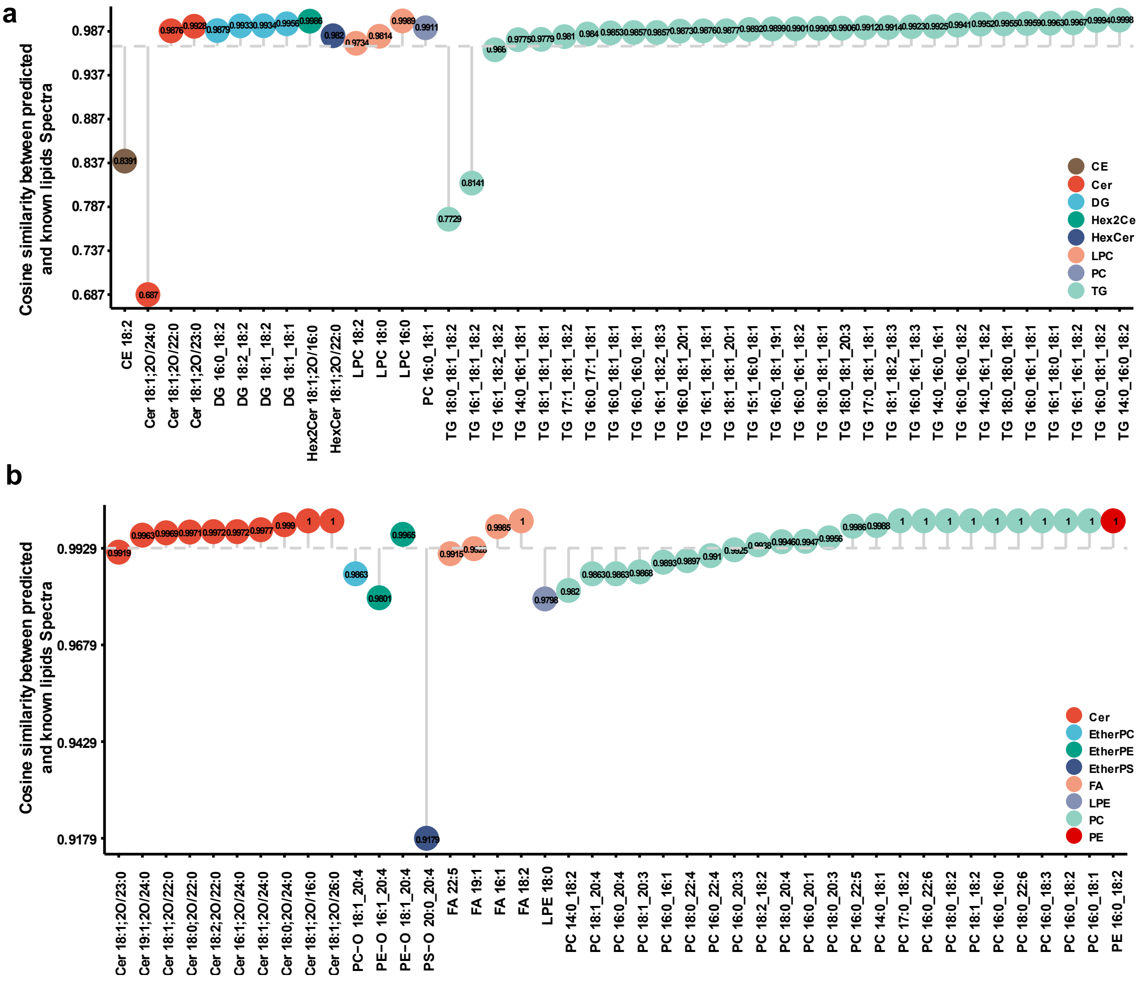

Supplementary Fig. 12. Dotted line plot of WMYn predicted NIST SRM 1950 spectrogram similarity using serum cohort 3 samples. a. Cosine similarity of 44 lipid profiles to known lipid profiles predicted using WMYn in positive ionization mode. **b.** Cosine similarity of 43 lipid profiles to known lipid profiles predicted using WMYn in negative ionization mode.

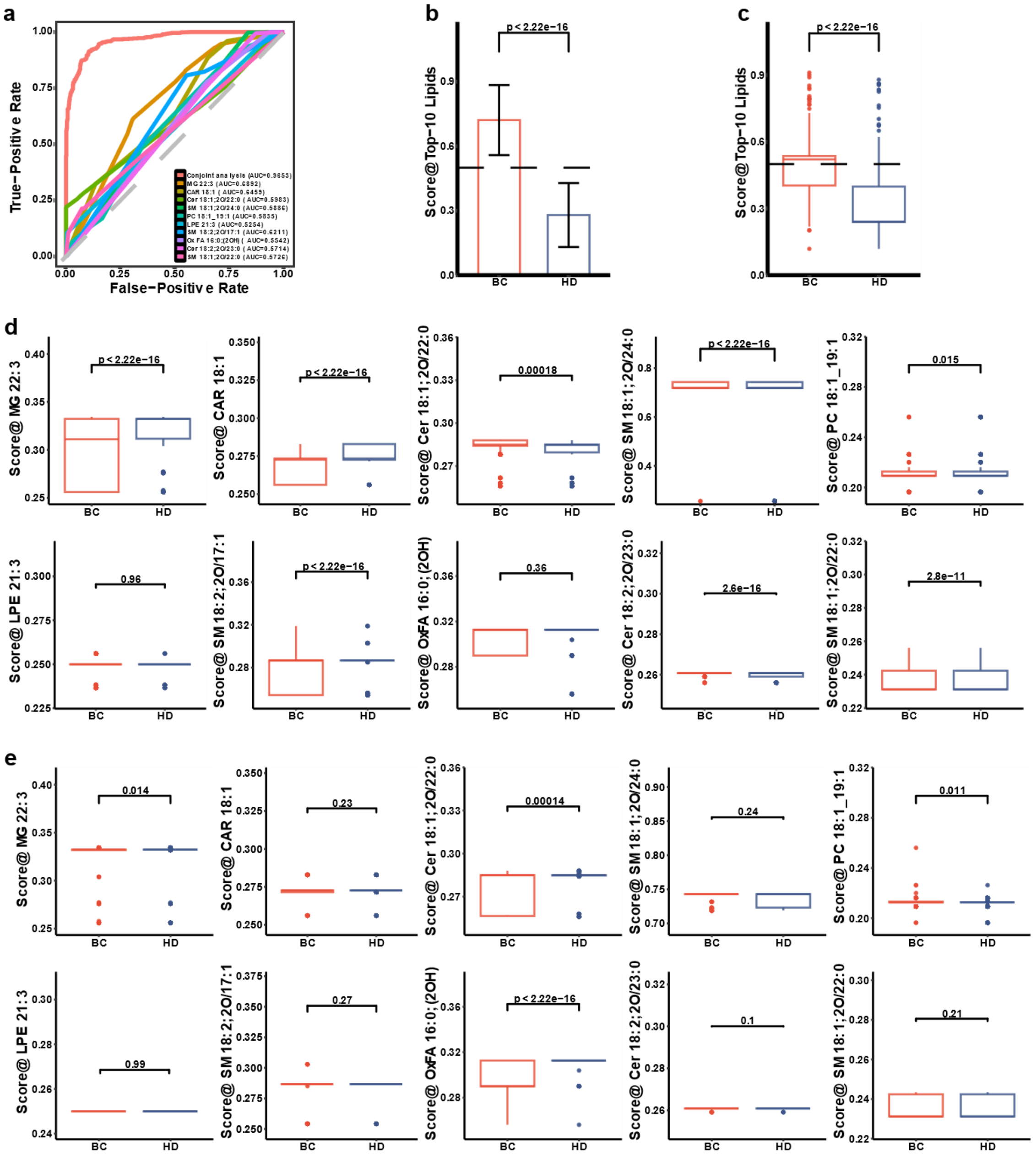
a
True−Positive Rate
False−Positive Rate
b
Score@Top−10 Lipids
p < 2.22e−16
BC
HD
c
Score@Top−10 Lipids
p < 2.22e−16
BC
HD
d
Score@ MG 22:3
p < 2.22e−16
Score@ CAR 18:1
p < 2.22e−16
Score@ Cer 18:1;2O/22:0
0.00018
Score@ SM 18:1;2O/24:0
p < 2.22e−16
Score@ PC 18:1_19:1
0.015
Score@ LPE 21:3
0.96
Score@ SM 18:2;2O/17:1
p < 2.22e−16
Score@ OxFA 16:0;(2OH)
0.36
Score@ Cer 18:2;2O/23:0
2.6e−16
Score@ SM 18:1;2O/22:0
2.8e−11
e
Score@ MG 22:3
0.014
Score@ CAR 18:1
0.23
Score@ Cer 18:1;2O/22:0
0.00014
Score@ SM 18:1;2O/24:0
0.24
Score@ PC 18:1_19:1
0.011
Score@ LPE 21:3
0.99
Score@ SM 18:2;2O/17:1
0.27
Score@ OxFA 16:0;(2OH)
p < 2.22e−16
Score@ Cer 18:2;2O/23:0
0.1
Score@ SM 18:1;2O/22:0
0.21

Supplementary Fig. 13. Differential analysis of selected biomarkers. a. The ROC curves demonstrate the effect of using biomarkers separately and in combination. **b.** Differences in scores of ten lipid markers in 1393 clinical samples using t-tests (two-sided), in the box plot the median as a center line, with the box representing the interquartile range (IQR) between the upper and lower quartiles. Whiskers extend to 1.5 times the IQR, and points beyond this range indicate outliers. **c.** Differences in scores of ten lipid markers in 333 clinical samples using t-tests (two-sided), in the box plot the median as a center line, with the box representing the interquartile range (IQR) between the upper and lower quartiles. Whiskers extend to 1.5 times the IQR, and points beyond this range indicate outliers. **d.** Differences in scores for each of the ten lipid markers in the 1393 clinical samples were examined separately using t-tests (two-sided), in the box plot the median as a center line, with the box representing the interquartile range (IQR) between the upper and lower quartiles. Whiskers extend to 1.5 times the IQR, and points beyond this range indicate outliers. **e.** Differences in scores for each of the ten lipid markers in the 333 clinical samples were examined separately using t-tests (two-sided), in the box plot the median as a center line, with the box representing the interquartile range (IQR) between the upper and lower quartiles. Whiskers extend to 1.5 times the IQR, and points beyond this range indicate outliers.

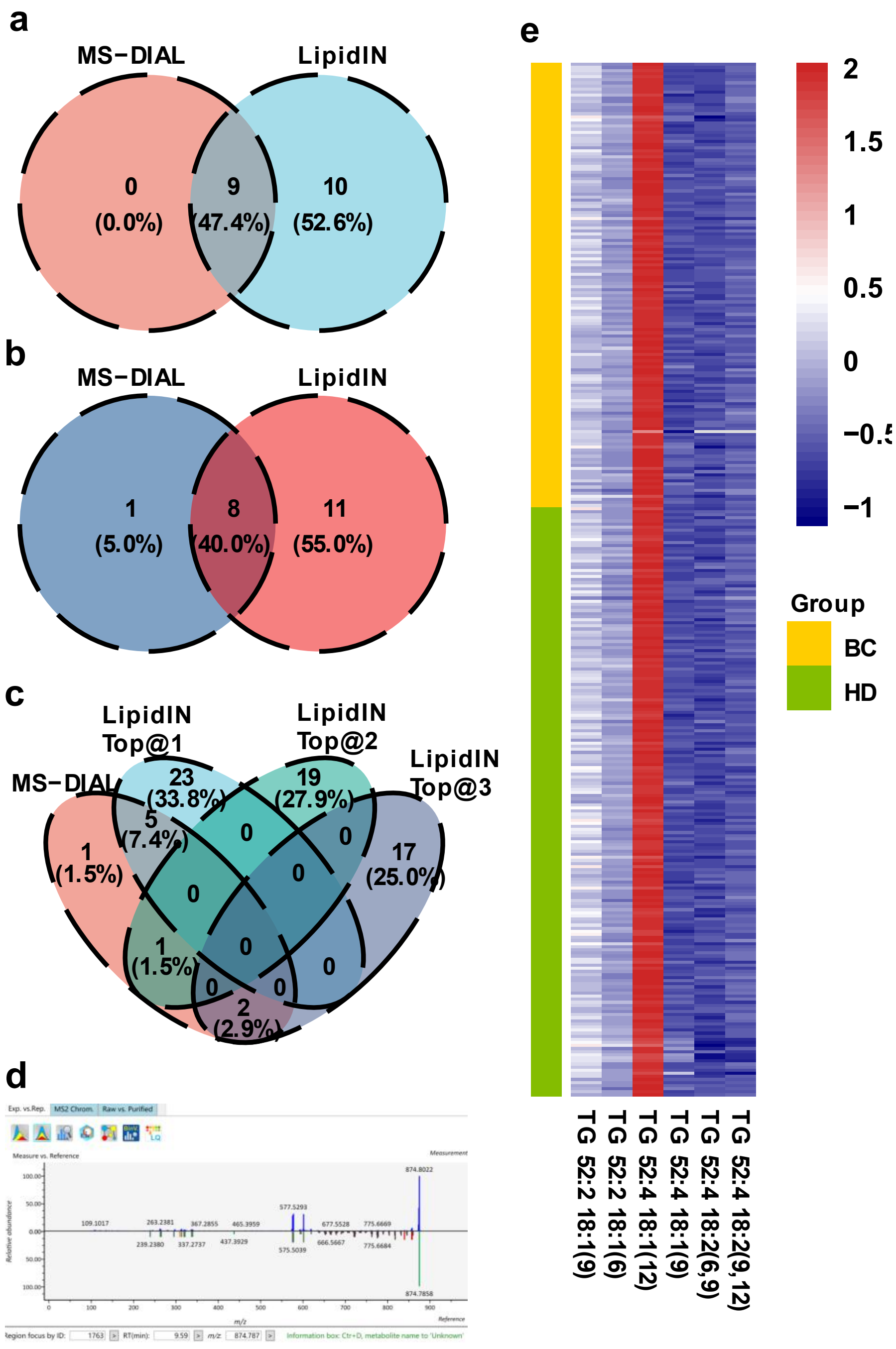

Supplementary Fig. 14. Annotated Performance and Clinical Case Study of LipidIN on the ZenoTOF 7600 System. a. Annotated intersections between LipidIN and MS-DIAL at the C:DB level. **b.** Annotated intersections between LipidIN and MS-DIAL at the fatty acid composition level. **c.** Annotated intersections between LipidIN and MS-DIAL at the C=C position level. **d.** Reference (blue) and measured (red) spectra of TG 16:0_16:1(9)_18:2(9,12). **e.** Analysis of differences in clinical data across 333 cases at specific double-bonded locations; BC indicates breast cancer, while HD indicates healthy donor.

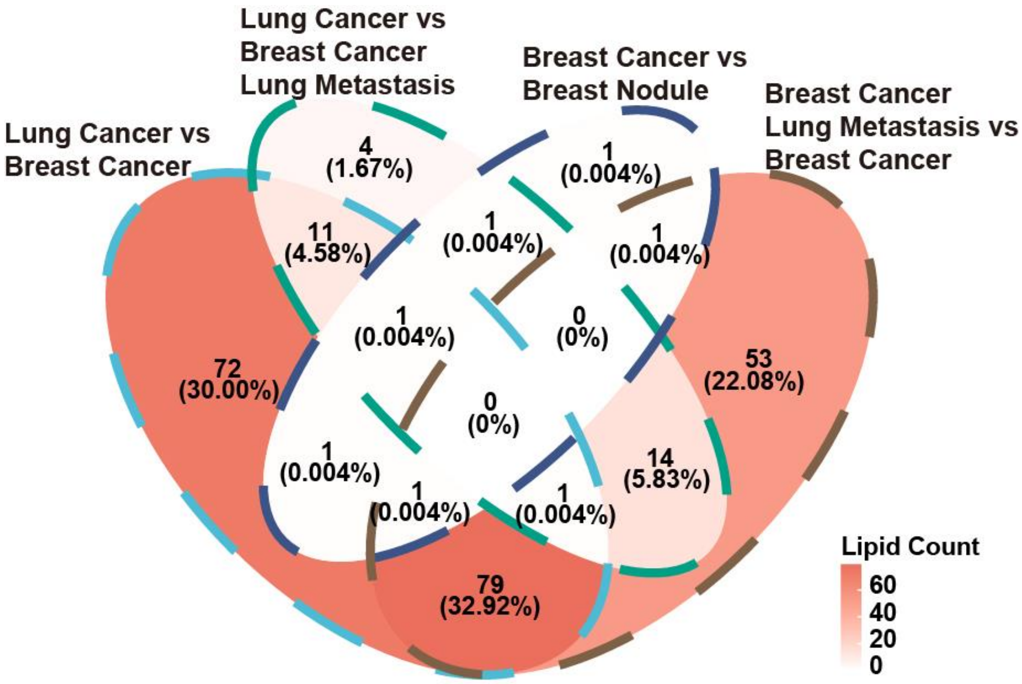

Supplementary Fig. 15. Potential biomarkers intersection for discriminating four groups with 1.4-fold change.

Supplementary Tables

Supplementary Table 1. Functional comparison of different tools.

Software name	Ion mobility data support	MS/MS similarity calculation	Decision tree annotation	Hierarchical library
LipidIN	Yes	Yes	Yes	Yes
MS-DIAL 5.1	Yes	Yes	Yes	No
LipidMatch 2.0.2	No	No	Yes	No
Entropy Search	No	Yes	No	No
LipidSearch 4.2	Yes	Yes	Yes	No

Supplementary Table 2. Parameters used in different tools.

Tool Names	MS1 tolerance	MS2 tolerance	Peak picking algorithms / packages	Version / Download data	other parameters
LipidIN	5 ppm / 10 ppm	10 ppm /20 ppm	RaMS	-	MS2_fliter: 0.01 Other Parameters Set to Default
MS-DIAL	0.01 Da	0.025 Da	Model-based peak detection	V 5.1.2	Other Parameters Set to Default
LipidMath	0.01 Da	0.025 Da	No peak detection	2.0.2	Other Parameters Set to Default
LipidSearch	5 ppm	10 ppm	Self-peak detection	V 4.2	Other Parameters Set to Default
Flash entropy	0.01 Da	0.025 Da	No peak detection	2023/9/23	Other Parameters Set to Default
Entropy Search	0.01 Da	0.025 Da	No peak detection	2024/5/3	Other Parameters Set to Default

Supplementary References

1. Murphy, R.C., Fiedler, J. & Hevko, J. Analysis of Nonvolatile Lipids by Mass Spectrometry. *Chemical Reviews* **101**, 479-526 (2001).
2. Giuffrida, F., Destaillats, F., Skibsted, L.H. & Dionisi, F. Structural analysis of hydroperoxy- and epoxy-triacylglycerols by liquid chromatography mass spectrometry. *Chem Phys Lipids* **131**, 41-49 (2004).
3. Merrill, A.H., Sullards, M.C., Allegood, J.C., Kelly, S. & Wang, E. Sphingolipidomics: High-throughput, structure-specific, and quantitative analysis of sphingolipids by liquid chromatography tandem mass spectrometry. *Methods* **36**, 207-224 (2005).
4. Domingues, M.R., Reis, A. & Domingues, P. Mass spectrometry analysis of oxidized phospholipids. *Chem Phys Lipids* **156**, 1-12 (2008).
5. Liu, X. et al. Oxidized fatty acid analysis by charge-switch derivatization, selected reaction monitoring, and accurate mass quantitation. *Anal Biochem* **442**, 40-50 (2013).
6. Ishibashi, Y. et al. A novel ether-linked phytol-containing digalactosylglycerolipid in the marine green alga, Ulva pertusa. *Biochem Biophys Res Commun* **452**, 873-880 (2014).
7. van der Hooft, J.J., Ridder, L., Barrett, M.P. & Burgess, K.E. Enhanced acylcarnitine annotation in high-resolution mass spectrometry data: fragmentation analysis for the classification and annotation of acylcarnitines. *Front Bioeng Biotechnol* **3**, 26 (2015).
8. Yang, K., Jenkins, C.M., Dilthey, B. & Gross, R.W. Multidimensional mass spectrometry-based shotgun lipidomics analysis of vinyl ether diglycerides. *Anal Bioanal Chem* **407**, 5199-5210 (2015).
9. Facchini, L., Losito, I., Cataldi, T.R. & Palmisano, F. Ceramide lipids in alive and thermally stressed mussels: an investigation by hydrophilic interaction liquid chromatography-electrospray ionization Fourier transform mass spectrometry. *J Mass Spectrom* **51**, 768-781 (2016).
10. Gee, P.T., Liew, C.Y., Thong, M.C. & Gay, M.C. Vitamin E analysis by ultra-performance convergence chromatography and structural elucidation of novel α-tocodienol by high-resolution mass spectrometry. *Food Chem* **196**, 367-373 (2016).
11. Lopalco, P., Stahl, J., Annese, C., Averhoff, B. & Corcelli, A. Identification of unique cardiolipin and monolysocardiolipin species in Acinetobacter baumannii. *Sci Rep* **7**, 2972 (2017).
12. Jackson, S.N. et al. AP-MALDI Mass Spectrometry Imaging of Gangliosides Using 2,6-Dihydroxyacetophenone. *J Am Soc Mass Spectrom* **29**, 1463-1472 (2018).
13. Mahmoodani, F. et al. Identification of Vitamin D3 Oxidation Products Using High-Resolution and Tandem Mass Spectrometry. *J Am Soc Mass Spectrom* **29**, 1442-1455 (2018).
14. Nuzzo, G. et al. UPLC⁻MS/MS Identification of Sterol Sulfates in Marine Diatoms. *Mar Drugs* **17** (2018).
15. Pham, T.H. et al. Targeting Modified Lipids during Routine Lipidomics Analysis using HILIC and C30 Reverse Phase Liquid Chromatography coupled to Mass Spectrometry. *Sci Rep* **9**, 5048 (2019).
16. Ding, J. et al. In-Silico-Generated Library for Sensitive Detection of 2-Dimethylaminoethylamine Derivatized FAHFA Lipids Using High-Resolution Tandem Mass Spectrometry. *Anal Chem* **92**, 5960-5968 (2020).
17. Kim, J. & Hoppel, C.L. Identification of unusual phospholipids from bovine heart

mitochondria by HPLC-MS/MS. *J Lipid Res* **61**, 1707-1719 (2020).
18. Linke, V. et al. A large-scale genome-lipid association map guides lipid identification. *Nat Metab* **2**, 1149-1162 (2020).
19. Long, N.P. et al. Advances in Liquid Chromatography–Mass Spectrometry-Based Lipidomics: A Look Ahead. *J Anal Test* **4**, 183-197 (2020).
20. Chao, H.C. & McLuckey, S.A. Manipulation of Ion Types via Gas-Phase Ion/Ion Chemistry for the Structural Characterization of the Glycan Moiety on Gangliosides. *Anal Chem* **93**, 15752-15760 (2021).
21. Sikorskaya, T.V., Efimova, K.V. & Imbs, A.B. Lipidomes of phylogenetically different symbiotic dinoflagellates of corals. *Phytochemistry* **181**, 112579 (2021).
22. Wang, H. et al. Comprehensive Lipidomic Analysis of Three Edible Brown Seaweeds Based on Reversed-Phase Liquid Chromatography Coupled with Quadrupole Time-of-Flight Mass Spectrometry. *J Agric Food Chem* **70**, 4138-4151 (2022).
23. Cudlman, L. et al. Characterization of Triacylglycerol Estolide Isomers Using High-Resolution Tandem Mass Spectrometry with Nanoelectrospray Ionization. *Biomolecules* **13** (2023).
24. Körber, T.T., Sitz, T., Abdalla, M.A., Mühling, K.H. & Rohn, S. LC-ESI-MS/MS Analysis of Sulfolipids and Galactolipids in Green and Red Lettuce (Lactuca sativa L.) as Influenced by Sulfur Nutrition. *Int J Mol Sci* **24** (2023).
25. Zhou, L. et al. Identification and discrimination of lilii bulbus origins based on lipidomics using UHPLC–QE-Orbitrap/MS/MS combined with chemometrics analysis. *J Food Compos Anal* **123**, 105512 (2023).
26. Chandramouli, A. & Kamat, S.S. A Facile LC-MS Method for Profiling Cholesterol and Cholesteryl Esters in Mammalian Cells and Tissues. *Biochemistry* **63**, 2300-2309 (2024).
27. Nicoll, C.R. et al. In vitro construction of the COQ metabolon unveils the molecular determinants of coenzyme Q biosynthesis. *Nat Catal* **7**, 148-160 (2024).
28. Qi, C. et al. Mass Spectrometry Imaging for the Characterization of C═C Localization in Unsaturated Lipid Isomers at the Single-Cell Level. *Anal Chem* **96**, 15085–15090 (2024).
29. Roman, D. et al. Structure Revision of a Widespread Marine Sulfonolipid Class Based on Isolation and Total Synthesis. *Angew Chem Int Ed Engl* **63**, e202401195 (2024).
30. Rudt, E., Schneider, S. & Hayen, H. Hyphenation of Liquid Chromatography and Trapped Ion Mobility - Mass Spectrometry for Characterization of Isomeric Phosphatidylethanolamines with Focus on N-Acylated Species. *J Am Soc Mass Spectrom* **35**, 1584-1593 (2024).
31. Singh, S. et al. PLD3 and PLD4 synthesize S,S-BMP, a key phospholipid enabling lipid degradation in lysosomes. *bioRxiv* (2024).
32. Zhou, Y. et al. Photoinduced Online Enrichment–Deglycosylation of Glycolipids for Enhancing Lipid Coverage and Identification in Single-Cell Mass Spectrometry. *Anal Chem* **96**, 17576-17585 (2024).
