# Supplementary material for: LipidIN: a comprehensive repository for flash platform-independent annotation and reverse lipidomics
Source: Nat Commun. 2025 May 16;16:4566. doi: 10.1038/s41467-025-59683-5 (PMC12084368; doi:10.1038/s41467-025-59683-5)
Supplement: Supplementary file 2 — Description of Additional Supplementary Files [file 41467_2025_59683_MOESM2_ESM.pdf]

Description of Additional Supplementary Files

File name: Supplementary Data 1
Description: Lipid subclasses in 5-level hierarchical library.

File name: Supplementary Data 2
Description: Published dataset used in Benchmark.

File name: Supplementary Data 3
Description: Negative ionization mode annotations of Flash entropy.

File name: Supplementary Data 4
Description: Flash entropy annotations of SM d-31:1.

File name: Supplementary Data 5
Description: Comparison of Lipid Categories Intelligence Modeling (LCI) and Lipid Data Analyzer (LDA) for removal of false positive annotations.

File name: Supplementary Data 6
Description: Manual checking results of uniquely annotations.

File name: Supplementary Data 7
Description: Manual Checked datasets from various samples of cells, mouse tissues, human sera and zebrafish tissues.

File name: Supplementary Data 8
Description: Lipid reference standards.

File name: Supplementary Data 9
Description: Querying speed in the 1726 clinical data.

File name: Supplementary Data 10
Description: Clinical information of 1393 cases in the first cohort.

File name: Supplementary Data 11
Description: Terminology and Definitions Summary.
