# Supplementary material for: LipidIN: a comprehensive repository for flash platform-independent annotation and reverse lipidomics
Source: Nat Commun. 2025 May 16;16:4566. doi: 10.1038/s41467-025-59683-5 (PMC12084368; doi:10.1038/s41467-025-59683-5)
Supplement: Supplementary file 4 — Reporting Summary [file 41467_2025_59683_MOESM4_ESM.pdf]

nature portfolio

Corresponding author(s): Shu-Hai Lin

Last updated by author(s): Mar 25, 2025

Reporting Summary

Nature Portfolio wishes to improve the reproducibility of the work that we publish. This form provides structure for consistency and transparency in reporting. For further information on Nature Portfolio policies, see our Editorial Policies and the Editorial Policy Checklist.

Statistics

For all statistical analyses, confirm that the following items are present in the figure legend, table legend, main text, or Methods section.

n/a	Confirmed	
☐	☒	The exact sample size (*n*) for each experimental group/condition, given as a discrete number and unit of measurement
☐	☒	A statement on whether measurements were taken from distinct samples or whether the same sample was measured repeatedly
☐	☒	The statistical test(s) used AND whether they are one- or two-sided<br>*Only common tests should be described solely by name; describe more complex techniques in the Methods section.*
☐	☒	A description of all covariates tested
☒	☐	A description of any assumptions or corrections, such as tests of normality and adjustment for multiple comparisons
☐	☒	A full description of the statistical parameters including central tendency (e.g. means) or other basic estimates (e.g. regression coefficient) AND variation (e.g. standard deviation) or associated estimates of uncertainty (e.g. confidence intervals)
☐	☒	For null hypothesis testing, the test statistic (e.g. *F*, *t*, *r*) with confidence intervals, effect sizes, degrees of freedom and *P* value noted<br>*Give P values as exact values whenever suitable.*
☒	☐	For Bayesian analysis, information on the choice of priors and Markov chain Monte Carlo settings
☒	☐	For hierarchical and complex designs, identification of the appropriate level for tests and full reporting of outcomes
☒	☐	Estimates of effect sizes (e.g. Cohen's *d*, Pearson's *r*), indicating how they were calculated

Our web collection on statistics for biologists contains articles on many of the points above.

Software and code

Policy information about availability of computer code

Data collection	The datasets in the three rule validations are from the following addresses, ST002384, ST001794 and ST003514 in Metabolomics Workbench (https://www.metabolomicsworkbench.org), DM0031 and DM0044 (https://prime.psc.riken.jp/menta.cgi/prime/drop_index), Metabolomics Workbench identifier MTBLS4684, MTBLS6965, MTBLS1369, MTBLS4654, and MTBLS6511 (https://www.ebi.ac.uk/metabolights/). Unless otherwise stated, all data supporting the results of this study are available in the article, supplementary materials, and source data files. Source data are provided with this article. The MS-DIAL published library was download at https://systemsomicslab.github.io/compms/msdial/main.html#MSP. Additional lipid hierarchical library calculated using an iterative algorithm with a total number of 168.5 million has been uploaded in zenodo (https://doi.org/10.5281/zenodo.14824498). MS data were convert to *.mzML with MSConvert v3.0, and MS system are list in Supporting Information Materials and Methods.
Data analysis	All benchmark tests were performed on a personal computer with 13th Gen Intel® Core™ i7-13700F × 16- Core Processor, 64 GB memory, and installed with Windows11 operation system , R-4.2.3 and Python v.3.9 including packages XCMS (v 4.2.2), RaMS (v 1.4.0), parallel (v 3.6.2), doParallel (version 1.0.17), Rcpp (1.0.11), tidyverse (v 1.3.0), WGCNA (v 1.7.0-3), statTarget (v 1.34.0), lightgbm (v 4.5.0), pandas (v 2.0.3.), numpy(v 1.23.5), torch (1.13.1+cu116). MS entropy and Flash entropy can be download from Github at https://github.com/YuanyueLi/SpectralEntropy, and https://github.com/YuanyueLi/FlashEntropySearch. LipidMatch can be downloaded from https://github.com/GarrettLab-UF/LipidMatch. In testing, we used MS-DIAL version v4.9.221218 and LipidSearch V4.2. All equations for LipidIN for MS/MS annotation and fingerprint regenerating are given in the Methods. In particular, MS-DIAL v 5.1 was used for TG double bond position annotation benchmark test for EAD systems. Code for clinical cohort analysis can be access at zenodo (hhttps://doi.org/10.5281/zenodo.14824498) and github (https://github.com/LinShuhaiLAB/LipidIN/Manuscript), and code ocean https://codeocean.com/capsule/3229548/tree.

For manuscripts utilizing custom algorithms or software that are central to the research but not yet described in published literature, software must be made available to editors and reviewers. We strongly encourage code deposition in a community repository (e.g. GitHub). See the Nature Portfolio guidelines for submitting code & software for further information.

Data

Policy information about availability of data

All manuscripts must include a data availability statement. This statement should provide the following information, where applicable:

- Accession codes, unique identifiers, or web links for publicly available datasets
- A description of any restrictions on data availability
- For clinical datasets or third party data, please ensure that the statement adheres to our policy

The mass spectrometry data of lipidomics were deposited to Metabolomics Workbench (https://www.ebi.ac.uk/metabolights/) and are accessible with identifier MTBLS10170 and also in National Genomics Data Center (https://ngdc.cncb.ac.cn/) PRJCA028507. TThe datasets in the three rule validations are from the following addresses, ST002384, ST001794 and ST003514 in Metabolomics Workbench (https://www.metabolomicsworkbench.org), DM0031 and DM0044 (https://prime.psc.riken.jp/menta.cgi/prime/drop_index), Metabolomics Workbench identifier MTBLS4684, MTBLS6965, MTBLS1369, MTBLS4654, and MTBLS6511 (https://www.ebi.ac.uk/metabolights/). The MS-DIAL published library was download at https://systemsomicslab.github.io/compms/msdial/main.html#MSP. Additional lipid hierarchical library calculated using an iterative algorithm with a total number of 168.5 million has been uploaded in zenodo (https://zenodo.org/records/14824498).

Research involving human participants, their data, or biological material

Policy information about studies with human participants or human data. See also policy information about sex, gender (identity/presentation), and sexual orientation and race, ethnicity and racism.

Reporting on sex and gender	We followed the sex and Gender Eauity in Research (SAGER) guideline and included the recommended information in our manuscript. This study was conducted with de-identified data of the participants who had consented to the use of their anonymized datain research. All the participants enrolled in this study were female patients.
Reporting on race, ethnicity, or other socially relevant groupings	We did not report detailed or specific race, ethnicity, or other socially relevant groupings for this study.
Population characteristics	We did not report detailed or specific population characteristics for this study. All the participants enrolled in this study were female patients, and they age range from 21 to 88. Body mass index range from 15.63 to 36.20. More detail is shown in Supplementary Data 10.
Recruitment	All participants were enrolled from Fujian Medical University Union Hospital between January 2018 and December 2022. Written informed consent was obtained from each study participant.
Ethics oversight	This study was performed in line with the principles of the Declaration of Helsinki. Approval was granted by the Ethics Committee of Fujian Medical University Union Hospital (Approval Number: 2022KY111) and Xiamen University Ethics Committee, Fujian Province, China (Approval number: XDYX202302K08).

Note that full information on the approval of the study protocol must also be provided in the manuscript.

Field-specific reporting

Please select the one below that is the best fit for your research. If you are not sure, read the appropriate sections before making your selection.

☒ Life sciences ☐ Behavioural & social sciences ☐ Ecological, evolutionary & environmental sciences

For a reference copy of the document with all sections, see nature.com/documents/nr-reporting-summary-flat.pdf

Life sciences study design

All studies must disclose on these points even when the disclosure is negative.

Sample size	Cohorts sample size was limited by number of subject in the study and number of samples collected from each subject. Our study does not involve life science experiments that require reproducibility validation.
Data exclusions	In LipidIN analysis, spectra without any fragment ions were excluded from analysis since they do not provide useful information for lipid annotation.<br>In clinical cohorts analysis, to ensure the accuracy and reliability of our analysis, we only included lipids in our lipidomics analysis that had a Scorematched of over 0.75 and a final score of 2.1 or higher in LipidIN annotations. When multiple plausible annotations existed under the same peak, we selected the one with the highest score. Furthermore, we excluded biological samples from our analysis if they had missing data exceeding 20%. Additionally, we removed lipids with coefficient of variation (CV) higher than 30% in quality control (QC) samples to maintain data consistency and reliability.
Replication	In lipid annotation, tests are performed using public datasets that have unambiguous annotation results for lipids. For the additionally annotations, we set the annotations that not only complied with the ECN rule in tolerance 0.5 min but also contained all feature peaks in high intensity to be correct.

	Biomarkers in the clinical cohort were confirmed by manual examination of spectra and retention time methods. In each tested sample group, we carefully adjusted the lipid intensity using internal standards as references.
Randomization	Participants were not randomized in this study. Samples were randomized for lipid extraction and mass spectrometry aquisition.
Blinding	There was no blinding in this study because it is not relevant to the study (there is no allocation to groups or interventions in the study). Omics data was processed without knowledge of participants' clinical status.

Reporting for specific materials, systems and methods

We require information from authors about some types of materials, experimental systems and methods used in many studies. Here, indicate whether each material, system or method listed is relevant to your study. If you are not sure if a list item applies to your research, read the appropriate section before selecting a response.

Materials & experimental systems

n/a	Involved in the study	
☒	☐	Antibodies
☒	☐	Eukaryotic cell lines
☒	☐	Palaeontology and archaeology
☒	☐	Animals and other organisms
☒	☐	Clinical data
☒	☐	Dual use research of concern
☒	☐	Plants

Methods

n/a	Involved in the study	
☒	☐	ChIP-seq
☒	☐	Flow cytometry
☒	☐	MRI-based neuroimaging

Plants

Seed stocks	*Report on the source of all seed stocks or other plant material used. If applicable, state the seed stock centre and catalogue number. If plant specimens were collected from the field, describe the collection location, date and sampling procedures.*
Novel plant genotypes	*Describe the methods by which all novel plant genotypes were produced. This includes those generated by transgenic approaches, gene editing, chemical/radiation-based mutagenesis and hybridization. For transgenic lines, describe the transformation method, the number of independent lines analyzed and the generation upon which experiments were performed. For gene-edited lines, describe the editor used, the endogenous sequence targeted for editing, the targeting guide RNA sequence (if applicable) and how the editor was applied.*
Authentication	*Describe any authentication procedures for each seed stock used or novel genotype generated. Describe any experiments used to assess the effect of a mutation and, where applicable, how potential secondary effects (e.g. second site T-DNA insertions, mosiacism, off-target gene editing) were examined.*
